# Somatic comorbidities of mental disorders in pregnancy

**DOI:** 10.1192/j.eurpsy.2023.1

**Published:** 2023-01-16

**Authors:** Vahe Khachadourian, Arad Kodesh, Stephen Z. Levine, Emma Lin, Joseph D. Buxbaum, Veerle Bergink, Sven Sandin, Abraham Reichenberg, Magdalena Janecka

**Affiliations:** 1 Department of Psychiatry, Icahn School of Medicine at Mount Sinai, New York, New York 10029, USA; 2 Seaver Autism Center for Research and Treatment, Icahn School of Medicine at Mount Sinai, New York, New York 10029, USA; 3 Mindich Child Health and Development Institute, Icahn School of Medicine at Mount Sinai, New York, New York 10029, USA; 4 The School of Public Health, University of Haifa, Haifa, Israel; 5 Meuhedet Health Services, Tel Aviv, Israel; 6 Friedman Brain Institute, Icahn School of Medicine at Mount Sinai, New York, New York 10029, USA; 7 Department of Obstetrics, Gynecology and Reproductive Science, Icahn School of Medicine at Mount Sinai, New York, New York 10029, USA; 8 Department of Psychiatry, Erasmus MC, Rotterdam, The Netherlands; 9 Department of Medical Epidemiology and Biostatistics, Karolinska Institutet, Stockholm, Sweden; 10 Department of Environmental Medicine and Public Health, Icahn School of Medicine at Mount Sinai, New York, New York 10029, USA; 11 Department of Genetic and Genomic Sciences, Icahn School of Medicine at Mount Sinai, New York, New York 10029, USA

**Keywords:** Comorbidity, maternal health, mental disorders, pregnancy, psychiatric epidemiology, somatic disorders

## Abstract

**Background:**

Mental and physical health conditions are frequently comorbid. Despite the widespread physiological and behavioral changes during pregnancy, the pattern of comorbidities among women in pregnancy is not well studied. This study aimed to systematically examine the associations between mental and somatic disorders before and during pregnancy.

**Method:**

The study used data from mothers of a nationally representative birth cohort of children born in Israel (1997–2008). We compared the risk of all major somatic disorders (International Classification of Diseases, Ninth Revision) in pregnant women with and without a mental disorder. All analyses were adjusted for maternal age, child’s birth year, family socioeconomic status, and the total number of maternal encounters with health services around pregnancy period.

**Results:**

The analytical sample included 77,030 mother–child dyads, with 30,083 unique mothers. The mean age at child’s birth was 29.8 years. Prevalence of diagnosis of mental disorder around pregnancy in our sample was 4.4%. Comorbidity between mental and somatic disorders was two times higher than the comorbidity between pairs of different somatic disorders. Of the 17 somatic disorder categories, seven were positively associated with mental health disorders. The highly prevalent comorbidities associated with mental disorders in pregnancy included e.g. musculoskeletal (OR = 1.30; 95% CI = 1.20–1.42) and digestive system diseases (OR = 1.23; 95% CI = 1.13–1.34).

**Conclusions:**

We observed that associations between maternal diagnoses and mental health stand out from the general pattern of comorbidity between nonmental health diseases. The study results confirm the need for screening for mental disorders during pregnancy and for potential comorbid conditions associated with mental disorders.

## Introduction

Multimorbidity, defined as co-occurrence of two or more diseases in the same person, is a major health problem affecting a substantial portion of the population [1]. Over the past decades, the prevalence of multimorbidity has been on the rise [2, 3]. Multimorbid health conditions negatively affect the quality of life of the patient, are costly to treat [4] and harder to manage than individual conditions [5, 6]. Moreover, the coexistence of multiple diseases may have health effects that are greater than the sum of the effects of individual diseases, which is especially problematic during pregnancy due to potential long-term adverse effects on both mother and the child [7–10].

Although the comorbidity between mental and somatic health conditions has received increasing attention over the past years [11, 12], still little is known about the full spectrum of mental and somatic comorbidities during pregnancy. Most studies have focused on specific pairs of health conditions, providing valuable insights about such comorbidity (e.g., depression and diabetes) [13, 14]. However, the comorbidity between a wider range of mental and somatic disorders in pregnancy has never been investigated systematically.

The knowledge about comorbidity between mental and somatic disorders from non-pregnant populations may not necessarily translate to pregnancy period. Pregnancy is a unique state with profound physiological and behavioral changes [15]. Women with pre-pregnancy chronic medical illness require special healthcare, because medication regimes and the natural course of mental and somatic disorders may change during this time. For example, pregnancy is a critical period of the onset of cardiovascular conditions, endocrine disorders, and blood diseases (e.g., hypertensive disorders of pregnancy, diabetes gravidarum, and anemia) [16–18]. In parallel, mood and anxiety disorders are highly prevalent in women in their reproductive ages, including during the perinatal period [19–21].

Both mental and somatic conditions during pregnancy have been associated with adverse outcomes in offspring [22, 23] (e.g., risk of infections, asthma, obesity, cognitive performance, and psychiatric disorders). There is an increasing awareness that many disorders may have at least partly fetal origins [24]. Consequently, an improved understanding of the comorbidity between mental and somatic disorders around the pregnancy period may not only offer novel directions into the diagnosis and management of maternal health disorders, but may also shed new light on the determinants of health outcomes for the child.

Using a nationally representative birth-cohort study, we investigate the spectrum of associations between maternal mental and *all* categories of somatic disorders in the period just before and during pregnancy—without assuming any causal relationships between these comorbidities. The overarching aims of the current study were to investigate the burden of somatic comorbidities in pregnant women with mental disorders, and examine the associations between mental and somatic disorders in pregnancy. Finally, we wanted to contextualize our findings by comparing the pattern of mental–somatic comorbidities to those that occur between somatic disorders.

## Materials and Methods

### Study design and population

We conducted a cohort study using a population-based sample from a large health maintenance organization (HMO) in Israel (Meuhedet), which has been described previously [25, 26]. Briefly, per legislation in Israel, citizens are required to obtain medical insurance from one of the existing HMOs that cannot prohibit a citizen joining on the grounds of socioeconomic status (SES), ethnicity, geographic location, health conditions, and health needs. The equivalent health plans and fee structure across HMOs, along with the regulations prohibiting HMOs from refusing a citizen membership, minimize the risk of ascertainment bias in our sample.

The cohort included all children born between January 1, 1997 and December 31, 2008, including (a) randomly selected 19.5% of all the births within the Meuhedet HMO during that period, and (b) all siblings of children selected in the first stage of the sampling who were also born 1997 to 2008.

All selected children were linked to their family records, creating mother–child dyads. Since our focus was on maternal rather than child’s health, pregnancies leading to multiple live births were represented by a single mother–child dyad in the analyses.

To assure ascertainment of maternal diagnosis during the 12 months preceding their pregnancies, the analytical sample was restricted to pregnancies leading to a live birth between January 1, 1999 and December 31, 2008. All dyads where maternal age was younger than 13, or older than 55, were excluded from the sample due to potential administrative errors in these records. All procedures contributing to this work comply with the ethical standards of the relevant national and institutional committees on human experimentation and with the Helsinki Declaration of 1975, as revised in 2008. The study protocol was reviewed and approved by the Helsinki Ethics Committee of the Meuhedet and the Institutional Review Board of the University of Haifa. Since the data did not include any individual identifiers, a waiver of informed consent was granted by the reviewing bodies.

### Somatic disorders

**Figure 1.**
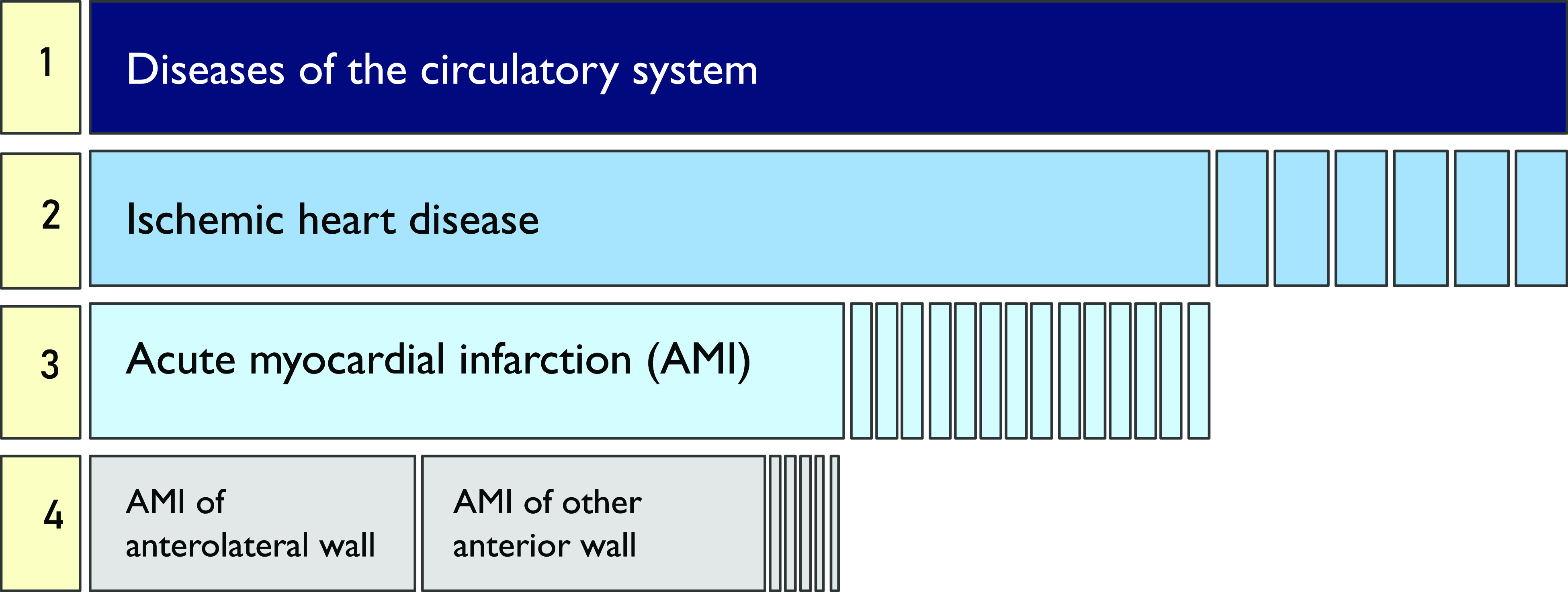
Example of the hierarchical organization of the ICD-9 taxonomy. ICD-9 categories are organized from the most general (level 1, top row), through most specific diagnostic codes (level 4, bottom row). Level 3 diagnoses were used in the current study.

The hierarchical organization of diagnostic codes in the International Classification of Diseases, Ninth Revision (ICD-9) has four levels, presenting information from least to most specific diagnosis (Figure 1). For the main analyses, *somatic disorders* were classified at level 1 ICD-9 codes using data from the Meuhedet Diagnostic Classification Register (e.g., ICD-9: 410.0, *acute myocardial infarction of anterolateral wall*, was classified with other *diseases of the circulatory system*; Figure 1). In additional analyses, we further defined more specific categories of somatic disorders, hereafter referred to as *specific somatic disorders*, according to diagnostic codes at level 3 (e.g., ICD-9: 410.0, *acute myocardial infarction of anterolateral wall*, was classified with other codes under *acute myocardial infarction*), as they offer an additional level of detail about the underlying health condition. The time window for ascertaining all diagnoses included a total of 636 days preceding the child’s birth, that is, the entire estimated pregnancy period (270 days), as well as the year preceding the conception, to maximize inclusion of chronic diagnoses in the analyses. Few diagnoses that were not coded according to ICD-9, and the available information did not allow for identifying and assigning them to any equivalent ICD-9, were omitted.

### Mental disorder

Using maternal diagnostic codes (per ICD-9) ascertained from the Meuhedet Diagnostic Classification Register, we classified general mental disorder (level 1), hereafter referred to as *mental disorder*, as the presence of any psychiatric diagnosis (ICD-9: 290–319, including all the subcodes) during the pregnancy period and the preceding year (i.e., the same time window as for the somatic disorders). While for the primary analyses, we pooled all diagnoses and only considered the maternal binary status (yes/no) of having any mental disorder during that time window, in additional analyses, we further defined more specific mental disorders, hereafter referred to as *specific mental disorders*, using the information from level 3 ICD-9 diagnostic codes, with a prevalence of at least 0.1% in the sample.

### Covariates

The covariates included maternal age at child’s birth, residential SES, and the total number of encounters with health services during the pregnancy period and the preceding year. Residential SES was a summary index based on household census data and was a function of the number of electrical appliances per person and per capita income in the area [27]. Information about SES was ascertained from Central Bureau of Statistics Registry, while the Meuhedet records served as the source for all other covariates.

To account for the varying risk of multiple diagnoses across the lifetime, the analysis adjusted for maternal age at child’s birth. The analyses also adjusted for SES to account for the differential likelihood of ascertaining maternal mental health [28] and other diagnoses [29] across the SES strata. Similarly, the categorized total number of encounters with health services (0–1, 2–5, 6–10, 11–15, 16–20, 21–30, 30+) during the 21-month period before child’s delivery was included in the adjusted analyses as a proxy measure for health-seeking behaviors, and healthcare utilization.

### Statistical analysis

#### Primary analysis

The associations between maternal mental and somatic disorders (all at ICD-9 level 1) were assessed using binary logistic regression models, where each somatic disorder served as an outcome in a separate, covariate-adjusted model. To account for potential clustering effects due to possible multiple deliveries among mothers in the study period, we used clustered sandwich estimator, implemented in the *clusterSEs* package (v2.6.2) [30].

Since the sampling strategy yielded a higher probability for the inclusion of mothers with multiple deliveries, we applied inverse probability selection weighting to account for these differential selection probabilities. The weights were computed based on the number of children born to each mother during the study period. Mothers with more offspring received lower weights in the regression model to account for their higher probability of inclusion in the sample (Supplementary Table S1).

To account for the possible correlation between somatic disorders—potentially driving some of the mental–somatic disorders associations, the initial analysis was followed by multivariable logistic regression models, which in addition to mental disorder and the covariates included all other level 1 somatic disorders other than the one serving as the outcome in that specific model.

#### Secondary analyses

We ran a series of secondary analyses to further inform the interpretation of the results from the primary analyses.

##### General comorbidity

First, we examined the association between pairs of somatic disorders, allowing us to put the burden of comorbidity between mental and somatic disorders (primary analysis) in the broader context of comorbidity. To this end, we evaluated the associations between all possible pairs of somatic disorders, adjusting for covariates.

##### Comorbidity patterns across specific mental disorders

Next, we examined whether specific mental disorders (level 3 ICD-9; e.g., “anxiety, dissociative and somatoform disorders” and “personality disorders”) were associated with different patterns of comorbidity. To this end, we repeated the analysis as specified for the primary analyses, using as an exposure each specific mental disorder with a prevalence of >0.1% in our analytical sample. These models included the same outcomes and covariates as described for the primary analysis.

##### Comorbidity of specific somatic disorders with mental disorder

Finally, we investigated whether the comorbidity patterns observed in our primary analysis are likely underlain by the association between mental disorder (ICD-9 level 1) and specific somatic disorders (ICD-9 level 3). Given the large number of specific somatic disorders, in these analyses we followed a systematic, multistep approach to minimize potential false-positive associations, including: (a) To address sparse data bias [31] all maternal specific somatic disorders with a recorded frequency of less than 10 in the pregnancies where mother either did, or did not receive a diagnosis for a mental disorder were excluded. (b) Each specific somatic disorder was assessed in a separate model (adjusted univariate models) that adjusted for maternal age, SES, year of birth, and total number of encounters with health services during the pregnancy period. (c) To address potential inflation of type I error due to multiple testing, *p*-values were corrected for a false discovery rate (*q*-value) of 5% [32]. (d) Each maternal somatic disorder that remained significantly associated with mental disorder after adjusting for multiple testing was evaluated in a model that was additionally adjusted for all the other maternal somatic disorder that remained significantly associated with mental disorder. Supplementary Figure S1 outlines the overview of the analytical strategy for this secondary analysis.

The robustness of our results in respect to the potential effect of missing data on study covariates were examined by sensitivity analyses comparing the results of complete case analyses with results obtained after multivariate imputation by chained equation [33]. We performed 10 imputations using information from all the variables in the model, including all level 1 diagnostic categories and covariates (maternal age at child’s birth, SES, and the total number of maternal diagnoses during the pregnancy period and the preceding year). All analyses were performed using R software (version 4.0.0) [34] including *mice*, *miceadds,* and *clusterSEs* packages.

## Results

**Figure 2.**
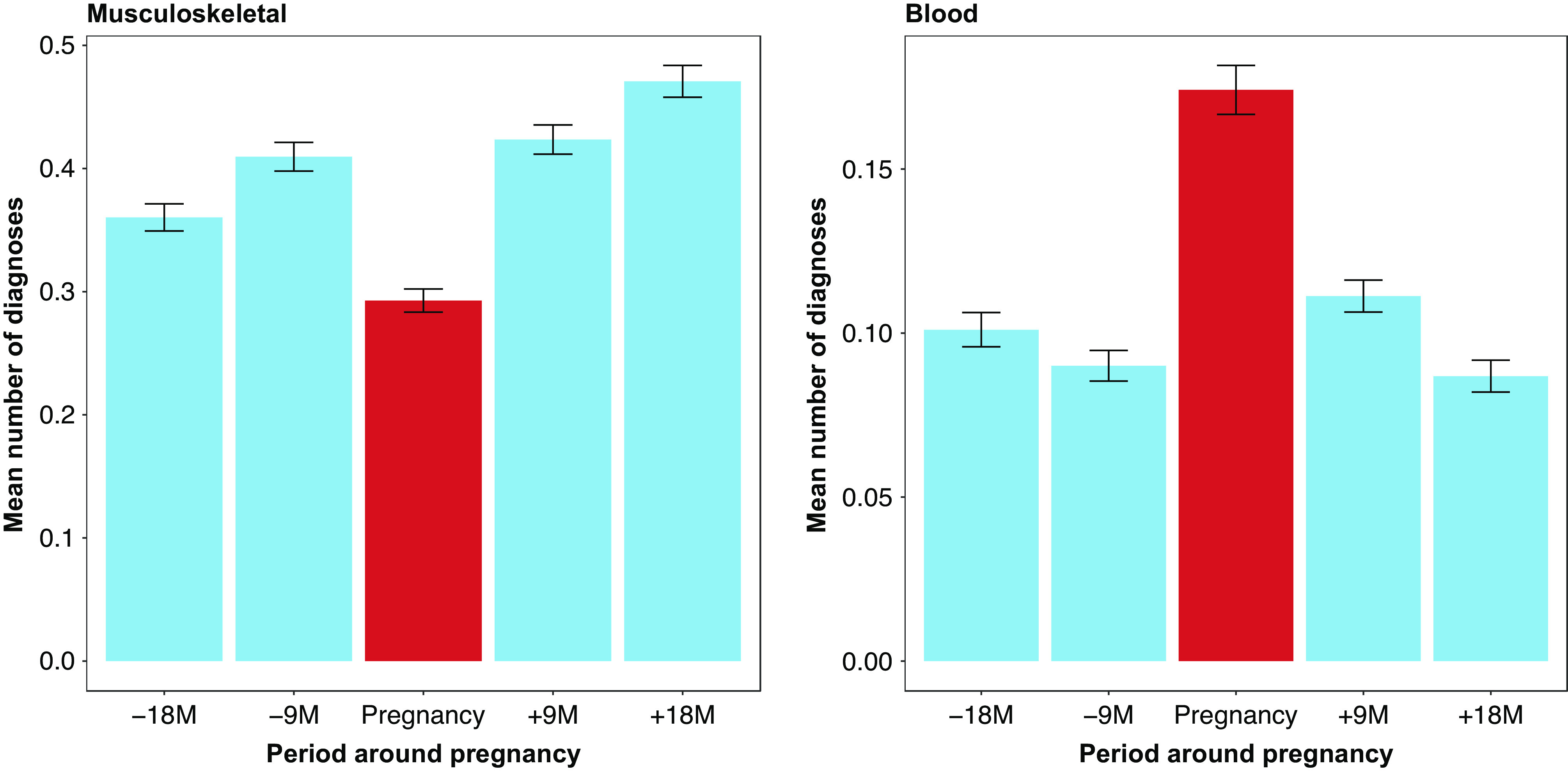
Mean number of diagnoses around pregnancy period by selected ICD-9 level 1 diagnostic categories (left: diseases of the musculoskeletal system and connective tissue, right: diseases of the blood and blood-forming organs).

The source population included 84,744 mother–child dyads, with children born 1999–2008. After removing the observations with missing values on SES (*n* = 7,699) and mother–child dyads with a maternal age at delivery below 13 or above 55 (*n* = 15), the analytical sample included 77,030 mother–child dyads, including 30,083 unique maternal IDs. None of the observations had a missing value for maternal age or child’s date of birth. Table 1 presents the analytical sample characteristics and prevalence of specific mental disorders with a minimum prevalence of 0.1%. In this population-based sample, pregnancy constituted a unique period with respect to the rates of diagnosis of majority of health conditions—with certain diagnoses (e.g., blood disorders) becoming more, and some less (e.g., musculoskeletal disorders) common in pregnancy, compared to the periods immediately before and after (Figure 2 and Supplementary Figure S2).

**Table 1.** Demographic characteristics of the analytical sample (mother–child dyads).

Variables	Any mental disorder (*n* = 3,361)	No mental disorder (*n* = 73,669)	Total (*N* = 77,030)
Maternal age at delivery, mean (SD)	31.4 (5.2)	29.7 (5.4)	29.8 (5.4)
Socioeconomic status (SES), mean (SD)	7.8 (4.3)	7.6 (4.3)	7.6 (4.3)
Delivery year, *n* (%)			
1999	243 (7.2%)	7,563 (10.3%)	7,806 (10.1%)
2000	289 (8.6%)	7,597 (10.3%)	7,886 (10.2%)
2001	291 (8.7%)	7,684 (10.4%)	7,975 (10.4%)
2002	320 (9.5%)	7,908 (10.7%)	8,228 (10.7%)
2003	308 (9.2%)	8,038 (10.9%)	8,346 (10.8%)
2004	352 (10.5%)	7,877 (10.7%)	8,229 (10.7%)
2005	436 (13.0%)	7,522 (10.2%)	7,958 (10.3%)
2006	379 (11.3%)	7,207 (9.8%)	7,586 (9.8%)
2007	467 (13.9%)	7,343 (10.0%)	7,810 (10.1%)
2008	276 (8.2%)	4,930 (6.7%)	5,206 (6.8%)
Total number of health encounters for somatic disorders, mean (SD)	29.2 (15.5)	18.6 (12.2)	19.1 (12.6)
Transient mental disorders due to conditions classified elsewhere (ICD-9: 293); *n* (%)			114 (0.1%)
Anxiety, dissociative and somatoform disorders (ICD-9: 300); *n* (%)			1,574 (2.0%)
Personality disorders (ICD-9: 301); *n* (%)			78 (0.1%)
Mental disorder related special symptoms or syndromes not elsewhere classified (ICD-9: 307); *n* (%)			948 (1.2%)
Depressive disorder, not elsewhere classified (ICD-9: 311); *n* (%)			633 (0.8%)

*Note:* Observations based on pregnancies; hence, mothers with multiple pregnancies contribute multiple data points.

**Figure 3.**
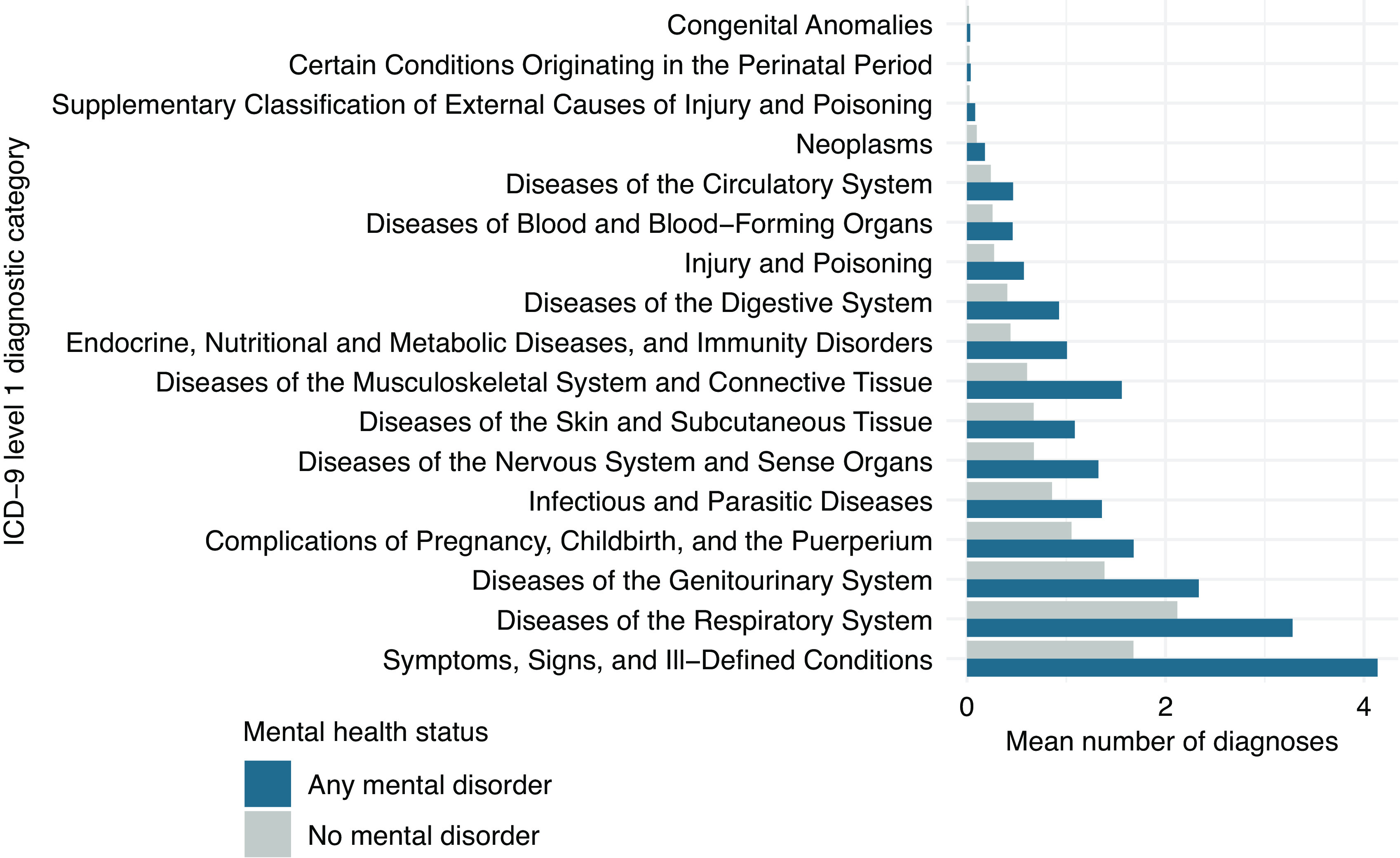
Mean number of somatic disorders (ICD-9 level 1 diagnosis) during the 21-month period before child’s delivery by mental health status.

Women with a mental disorder had a substantially higher number of health encounters for somatic disorders (including for chronic/recurrent disorders) recorded around pregnancy, compared to women without any mental disorders (*p* < 0.001). Presence of mental disorder was associated with a higher number of diagnosis from all categories of somatic disorders (Figure 3).

### Associations between maternal mental and somatic disorders

**Figure 4.**
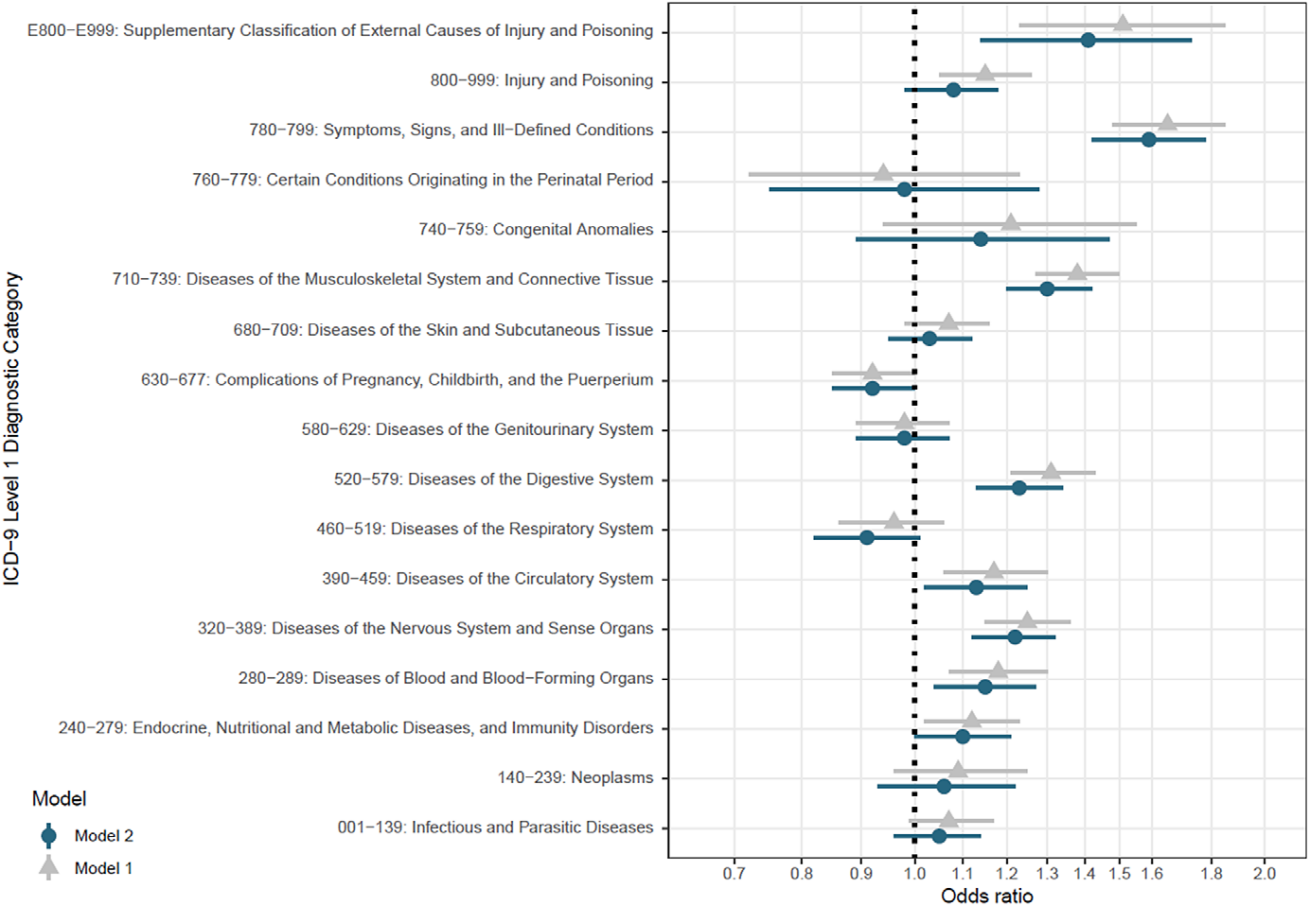
Associations between maternal somatic and mental disorders. Model 1 was adjusted for SES, maternal age at delivery, total number of encounters with health services during the 21 months period before delivery, and year of delivery. Model 2 was adjusted for all variables in model one as well as all the somatic disorder categories presented in this figure.

The associations between maternal mental disorder and all categories of somatic disorders around pregnancy are presented in Figure 4 and Supplementary Table S2. Maternal mental disorder was statistically significantly associated with an increased risk of 8 out of 17 somatic disorder categories. Maternal mental disorder around pregnancy was most strongly associated with diseases of the musculoskeletal system and connective tissue (OR = 1.30; 95% CI = 1.20, 1.42), digestive system diseases (OR = 1.23; 95% CI = 1.13–1.34), and neurological diseases (OR = 1.22; 95% CI = 1.12–1.32). Interrogating the risk of receiving a diagnosis within these categories of somatic disorders in association with specific mental disorders (e.g., depression and personality disorders), we observed a similar pattern of associations (see section “*Comorbidity patterns across specific mental disorders*” of the Supplementary Material). Detailed results on the associations of mental disorder with specific somatic disorders are presented in Section “*Comorbidity of specific somatic disorders with mental disorder*” of the Supplementary Material.

### General comorbidity

Comparative analyses exploring the relationship between all the possible pairs of somatic disorders (level 1) yielded several statistically significant associations (Table 2). On average, each somatic disorder category was positively associated with 3.5 other disorders (somatic and mental disorders), whereas mental disorders were positively associated with 7.0 somatic disorders. Higher comorbidity rate for mental disorders was observed in comparison with both rare and prevalent somatic disorders, indicating that our results were not due to differential statistical power to detect associations. Some of the observed associations between somatic diagnoses were of small magnitude, which despite being statistically significant (due to a high power) could lack clinical relevance.

**Table 2.** Adjusted odds ratio of the associations between mental and somatic disorders.

		Exposure (ICD-9 level 1 diagnostic category)																	
		MH	C17	C16	C15	C14	C13	C12	C11	C10	C9	C8	C7	C6	C5	C4	C3	C2	C1
Outcome ICD-9 level 1 diagnostic category)	C1	1.05	0.89	1.07**	1.09***	0.95	1.03	1.01	1.41***	0.93***	1.00	1.12***	1.27***	1.09**	1.10***	0.94*	0.97	1.18***	*N/A*
	C2	1.06	0.99	1.03	0.94	0.91	1.64***	0.94	1.75***	0.82***	1.08	0.97	0.85***	1.27***	1.14***	0.98	1.12*	*N/A*	
	C3	1.10	0.81*	0.94	1.15***	0.90	1.06	0.98	0.94*	1.09**	0.91**	1.01	0.84***	1.10**	0.98	1.36***	*N/A*		
	C4	1.15*	0.98	0.92*	1.21***	0.96	0.93	1.00	1.09**	1.31***	1.06	1.17***	0.95	1.15***	0.95	*N/A*			
	C5	1.22***	0.90	1.12***	1.09***	0.86*	1.04	1.12***	1.10***	0.90***	0.90***	1.15***	1.22***	1.01	*N/A*				
	C6	1.13*	0.99	0.99	1.07*	1.03	1.17	1.12***	1.10***	1.26***	0.91**	1.28***	1.03	*N/A*					
	C7	0.91	0.87	1.10***	1.29***	0.72***	1.16	1.12***	0.99	0.82***	0.86***	1.20***	*N/A*						
	C8	1.23***	0.92	1.07*	1.57***	0.95	1.13	1.22***	1.08**	0.97	1.04	*N/A*							
	C9	0.98	0.86*	0.89***	1.16***	0.96	0.99	1.02	0.97	1.12***	*N/A*								
	C10	0.92	1.02	0.94*	1.04	1.42***	1.12	0.91***	0.90***	*N/A*									
	C11	1.03	0.99	1.27***	0.96	0.88	1.30***	1.08**	*N/A*										
	C12	1.30***	1.96***	1.44***	1.36***	0.87	1.40***	*N/A*											
	C13	1.14	0.84	1.21	1.00	0.99	*N/A*												
	C14	0.98	0.54	1.01	0.99	*N/A*													
	C15	1.59***	1.04	1.09**	*N/A*														
	C16	1.08	4.12***	*N/A*															
	C17	1.41**	*N/A*																
	MH	*N/A*																	

*Note:* Odds ratios are adjusted for SES, maternal age at delivery, total number of encounters with health services during the 21 months period before delivery, year of delivery, and all the somatic and mental disorders presented in this table. Statistically significant positive associations are highlighted in blue and negative associations are highlighted in yellow.

Abbreviations: C1, infectious and parasitic diseases; C2, neoplasms; C3, endocrine, nutritional and metabolic diseases, and immunity disorders; C4, diseases of blood and blood-forming organs; C5, diseases of the nervous system and sense organs; C6, diseases of the circulatory system; C7, diseases of the respiratory system; C8, diseases of the digestive system; C9, diseases of the genitourinary system; C10, complications of pregnancy, childbirth, and the puerperium; C11, diseases of the skin and subcutaneous tissue; C12, diseases of the musculoskeletal system and connective tissue; C13, congenital anomalies; C14, certain conditions originating in the perinatal period; C15, symptoms, signs, and ill-defined conditions; C16, injury and poisoning; C17, supplementary classification of external causes of injury and poisoning; MH, mental disorders.

*q-value <0.05.

**q-value <0.01.

***q-value <0.001.

Sensitivity analysis using multiple imputation of missing variables yielded results that were near-identical to ones observed in complete case analyses, suggesting our findings were robust to the missingness pattern present in our dataset (Supplementary Table S3).

## Discussion

Our study adds to a body of literature showing that mental disorder around pregnancy is associated with a host of somatic comorbidities. Remarkably, the burden of somatic comorbidities associated with mental disorders was two times higher than that associated with another somatic disorder. This finding is novel and clinically relevant, as it indicates a higher co-occurrence between mental disorders and somatic complications in pregnancy relative to other types of comorbidites.

In this study, we systematically investigated the associations of somatic disorders around pregnancy (12 months before and during pregnancy) with maternal mental disorder. Initial analyses identified 10 broad somatic disorder categories, including musculoskeletal, neurological, and digestive system diseases, that occur at a higher rate in women with mental disorders in the antenatal period, compared to those without any mental disorder. Further analyses of over 700 specific somatic disorders grouped under those broader categories pointed to more specific associations (e.g. between mental disorders and hypertensive diseases), consistent with the existing literature about comorbidity and co-occurrence of somatic and mental disorders [35, 36].

In addition to the already well-established associations, our analyses yielded indications for potentially novel or less commonly studied associations. The somatic disorders most strongly associated with mental disorder included musculoskeletal, neurological, and digestive system diseases. Previous studies have found co-occurrence of mental and musculoskeletal disorders in working age population and aging women [37–39]. In our study, we observed that a similar pattern of comorbidity exists before and during pregnancy. Similarly, we observed an association between neurological diseases and mental disorders, which have been previously shown to be bidirectionally related [40]. Studies have also demonstrated higher rates of anxiety and depression among individuals suffering from irritable bowel syndrome and ulcerative colitis [41]. In line with these findings, we observed a positive association between digestive system diseases and mental disorders. Although we observed a strong association between mental disorders and symptoms, signs, and ill-defined conditions, this was not unexpected as many mental disorders have somatic manifestations which can be part of their diagnostic criteria [42]. Our results from the secondary analyses pointed to novel associations between mental disorders and lacrimal system disorders, and disorders of fluid, electrolyte, and acid–base balance which can serve as the basis for developing new hypotheses.

Importantly, our data present no support for the hypothesis that the patterns of somatic comorbidities vary between different mental disorders. Although the measures of associations between somatic disorders and personality disorders were not statistically significant (at least partly due to the low prevalence of personality disorders in the study sample), their direction and magnitude were mainly consistent with the direction and magnitude of the observed associations between somatic and other specific mental disorders.

The study results confirm the need for screening for mental disorders during pregnancy, a practice recommended by the obstetric guidelines of the American Obstetric Association [43]. Clinicians should also be aware of the high co-occurrence of somatic diseases (e.g., neurological, gastrointestinal, and musculoskeletal diseases) among patients with mental health diagnosis as comorbidities can worsen the course of disease for all the diseases that are present [10, 44]. For researchers, this study can offer insights into the complex network of associations between maternal health conditions and their impact on pregnancy and child outcomes. From the methodical perspective, our results can also inform selection of confounding variables in studies assessing impact of certain maternal diagnoses on health outcomes in the offspring. Even in situations when data on these measures are not available, pervasive nature of comorbidity should be acknowledged when drawing inferences from associations between maternal health and offspring outcomes.

### Strengths and limitations

The rigor and systematic nature of our approach should not be inferred as evidence for causality between maternal somatic and mental disorders. Although overall, our findings are consistent with the existing literature about high comorbidity between mental and somatic disorders [35, 36], the underlying mechanisms of the observed associations in our sample remain to be explored. While the presence of a mental disorder could cause somatic disorder (and vice-versa), shared underlying mechanisms should also be considered. Teasing out causal relationships will require a different methodology and approach, including careful consideration of confounding variables and establishing temporality between somatic and mental disorders. Since the time window for diagnosis of mental and somatic disorders was defined as pregnancy period and 1 year preceding it, the chronic conditions, particularly those adequately managed without requiring frequent health care visits, were less likely to be ascertained. Although we had adequate power to analyze associations between multiple somatic and mental disorders, there were less-common somatic disorders that were not included due to having a frequency of less than 10 across the mothers with or without mental disorders. Although defining the exposure based on at least one record of the specific diagnosis codes offered a higher sensitivity for identifying maternal diagnoses, it might have affected our specificity (e.g., due to administrative errors, or misdiagnosis). The study had limited data for covariate control. Lack of data on routinely adjusted health behaviors, including smoking, alcohol use, and physical activity did not allow us to investigate the possible behavioral factors that could lead to the observed associations between certain pairs of disorders. Additionally, the SES measure in our dataset was based on residential SES and it did not include household or individual level information. Although, we adjusted for residential SES, it could not capture the full depth of individual and household SES, [45, 46] therefore the SES adjustment in our analyses should be deemed incomplete. The analyses adjusted for the total number of encounters with health services during the 21-month period before delivery, which to a certain extent can account for differential health care access and utilization.

## Conclusions

In conclusion, this study followed a novel approach, supplementing prior research [47] with new insights on associations between a wide range of maternal somatic and mental health diagnoses around pregnancy. We demonstrated the high burden of somatic comorbidities among pregnant women with a diagnosis of mental health disorder, especially musculoskeletal, neurological, and digestive system diseases. The degree of comorbidity was twice as high compared to other pairs of somatic health conditions. Women with mental health problems are at high risk of “no shows” and having less control visits puts them at risk for missing somatic diagnoses. Moreover, their mental health conditions can make it difficult to adequately diagnose and monitor their somatic health, and treat comorbid somatic conditions. Awareness of the high comorbidity rates between mental and somatic disorders is thus of critical importance.

## Data Availability

Data access rules do not permit public sharing of the data. Interested researchers should discuss access options with A.K. and S.Z.L.

## Supplementary material

For supplementary material accompanying this paper visit http://doi.org/10.1192/j.eurpsy.2023.1. click here to view supplementary material
