# Supplementary material for: Somatic comorbidities of mental disorders in pregnancy
Source: Eur Psychiatry. 2023 Jan 16;66(1):e15. doi: 10.1192/j.eurpsy.2023.1 (PMC9970155; doi:10.1192/j.eurpsy.2023.1)
Supplement: Supplementary file 1 [file S0924933823000019sup001.docx]

**SUPPLEMENTARY MATERIALS**

Table of Contents

[Comorbidity patterns across different mental disorders 2](#_Toc95481184)

[Comorbidity of specific somatic disorders with mental disorder 3](#_Toc95481185)

[Figure S1. 4](#_Toc95481186)

[Figure S2. 5](#_Toc95481187)

[Figure S3. 6](#_Toc95481188)

[Table S2. 8](#_Toc95481189)

[Table S3. 9](#_Toc95481190)

[Table S4. 10](#_Toc95481191)

[Table S6. 12](#_Toc95481192)

# **Comorbidity patterns across specific mental disorders**

The frequencies of specific somatic disorders among women with and without specific mental disorders are presented in **Table S3**. We found that different specific mental disorders (e.g., depression, personality disorders) were mainly associated with similar somatic disorder categories (**Table S4**). For example, ICD-9 category of Diseases of Blood and Blood-Forming Organs was associated with both anxiety, dissociative, and somatoform disorders (OR = 1.21; 95% CI = 1.05-1.39), and depressive disorder (OR = 1.34; 95% CI = 1.08-1.66)). The direction of the estimated effects on different somatic disorders was generally consistent across different specific mental disorders - even for pairs of diagnoses that did not reach statistical significance, with strongly overlapping confidence intervals. These associations were also similar to the pattern recorded in the primary analysis, i.e., estimating risk of different somatic disorders associated with having any mental disorder (**Table S2**).

# **Comorbidity of specific somatic disorders with mental disorder**

There was a total of 786 distinct level 3 ICD-9 diagnostic codes in our sample, including 27 specific mental and 759 specific somatic diagnoses. After excluding specific somatic disorders recorded in <10 women with, or <10 women without, comorbid mental disorder in pregnancy, we retained 261 specific somatic disorder categories. In a series of logistic regression models adjusting for covariates and multiple testing, 52 of the 261 specific somatic disorders (20%) were statistically significantly associated (47 positive associations and 5 negative associations) with receiving any mental disorder diagnosis (**Table S6**). **Table S6** presents all coefficient estimates with their corresponding 95% confidence intervals, q-values (p-values adjusted for false discovery rate), and frequency of specific somatic disorders among those with and without any mental disorder. In a logistic regression model evaluating the associations of each of those 52 specific somatic disorders with mental disorder while also adjusting for the remaining 51 specific somatic disorders and other covariates, 37 associations remained statistically significant (**Table S7**).


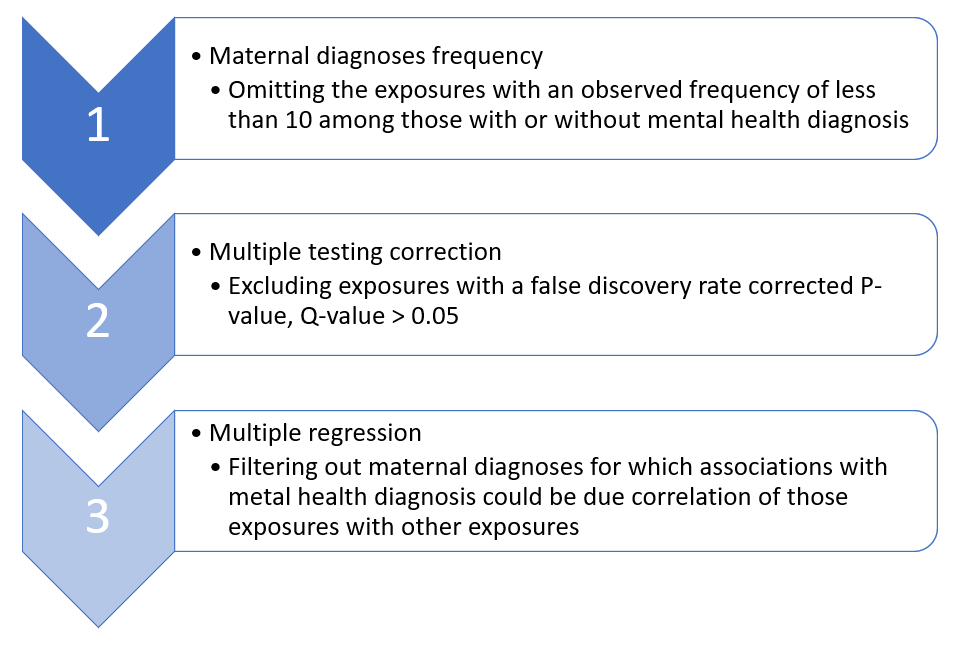


**Figure S1.** Outline of analytical steps for filtering out potential false positive associations between maternal mental and somatic disorders

**Figure S2.** Mean number of diagnoses around pregnancy period by selected somatic disorder categories

**Table S1.** Family size-based weights reflecting inverse probability of being included in the study.

| **Number of Children** | **Probability of being included in the sample** | **Unstandardized weights** | **Standardized weights** |
| --- | --- | --- | --- |
| **1** | 0.195 | 5.128 | 2.374 |
| **2** | 1 - (1 - 0.195)^2^ | 2.841 | 1.315 |
| **3** | 1 - (1 - 0.195)^3^ | 1.853 | 0.858 |
| **4** | 1 - (1 - 0.195)^4^ | 1.364 | 0.632 |
| **≥ 5** | ~ 1 - (1 - 0.195)^5^ | 1.078 | 0.499 |

**Table S2.** Associations between maternal ICD-9 level 1 diagnostic categories and ICD-9 level 1 mental health diagnosis

|  | **Univariate adjusted logistic regression model^a^** | **Multivariable logistic regression model^b^** | **Exposed** | | | |
| --- | --- | --- | --- | --- | --- | --- |
|  |  |  | **Any mental health diagnosis (ICD-19: 290-319), N= 3353** | | **No mental health diagnosis, N= 73423** | |
| **Level 1 ICD-9 diagnostic category** | **OR (95%CI)** | **OR (95%CI)** | **n** | **%** | **n** | **%** |
| **001-139:** Infectious and Parasitic Diseases | 1.07 (0.99, 1.17) | 1.05 (0.96,1.14) | 1916 | 57.0% | 30671 | 41.6% |
| **140-239:** Neoplasms | 1.09 (0.96, 1.25) | 1.06 (0.93,1.22) | 358 | 10.7% | 4830 | 6.6% |
| **240-279:** Endocrine, Nutritional and Metabolic Diseases, and Immunity Disorders | 1.12 (1.02, 1.23)* | 1.10 (1.00,1.21) | 916 | 27.3% | 11166 | 15.2% |
| **280-289:** Diseases of Blood and Blood-Forming Organs | 1.18 (1.07, 1.30)** | 1.15 (1.04,1.27)* | 756 | 22.5% | 10056 | 13.7% |
| **320-389:** Diseases of the Nervous System and Sense Organs | 1.25 (1.15, 1.36)*** | 1.22 (1.12,1.32)*** | 1705 | 50.7% | 24368 | 33.1% |
| **390-459:** Diseases of the Circulatory System | 1.17 (1.06, 1.30)** | 1.13 (1.02,1.25)* | 743 | 22.1% | 9678 | 13.1% |
| **460-519:** Diseases of the Respiratory System | 0.96 (0.86, 1.06) | 0.91 (0.82,1.01) | 2668 | 79.4% | 47782 | 64.9% |
| **520-579:** Diseases of the Digestive System | 1.31 (1.21, 1.43)*** | 1.23 (1.13,1.34)*** | 1367 | 40.7% | 16785 | 22.8% |
| **580-629:** Diseases of the Genitourinary System | 0.98 (0.89, 1.07) | 0.98 (0.89,1.07) | 2183 | 65.0% | 35161 | 47.7% |
| **630-677:** Complications of Pregnancy, Childbirth, and the Puerperium | 0.92 (0.85, 1.00) | 0.92 (0.85,1.00) | 1732 | 51.5% | 28867 | 39.2% |
| **680-709:** Diseases of the Skin and Subcutaneous Tissue | 1.07 (0.98, 1.16) | 1.03 (0.95,1.12) | 1551 | 46.1% | 24355 | 33.1% |
| **710-739:** Diseases of the Musculoskeletal System and Connective Tissue | 1.38 (1.27, 1.50)*** | 1.30 (1.20,1.42)*** | 1674 | 49.8% | 20737 | 28.1% |
| **740-759:** Congenital Anomalies | 1.21 (0.94, 1.55) | 1.14 (0.89,1.47) | 87 | 2.6% | 1039 | 1.4% |
| **760-779:** Certain Conditions Originating in the Perinatal Period | 0.94 (0.72, 1.23) | 0.98 (0.75,1.28) | 75 | 2.2% | 1320 | 1.8% |
| **780-799:** Symptoms, Signs, and Ill-Defined Conditions | 1.65 (1.48, 1.85)*** | 1.59 (1.42,1.78)*** | 2776 | 82.6% | 41762 | 56.7% |
| **800-999:** Injury and Poisoning | 1.15 (1.05, 1.26)** | 1.08 (0.98,1.18) | 908 | 27.0% | 12081 | 16.4% |
| **E800-E999:** Supplementary Classification of External Causes of Injury and Poisoning | 1.51 (1.23, 1.85)*** | 1.41 (1.14,1.73)** | 143 | 4.3% | 1296 | 1.8% |
| ^a^ models adjusted for SES, maternal age at delivery, total number of diagnoses in pregnancy, year of delivery  ^b^ model adjusted for SES, maternal age at delivery, total number of encounters with health services during 21 months period before delivery, year of delivery, and all the somatic disorders presented in this table; Statistically significant positive associations are highlighted in blue.  *: Q-value <0.05; **: Q-value <0.01; ***: Q-value <0.001 | | | | | | |

**Table S3.** Comparison of results obtained using (1) logistic regression (no imputation) and (2) logistic regression with imputation, for the associations between maternal somatic and mental disorders

|  | **Multivariable logistic regression model^a^ no imputation (N=77,030)** | **Multivariable logistic regression model^a^ with imputation (N=84,729)** | |
| --- | --- | --- | --- |
| **Somatic disorders (level 1 ICD-9 diagnostic category)** | **OR (95%CI)** | **OR (95%CI)** | |
| **001-139:** Infectious and Parasitic Diseases | 1.05 (0.96,1.14) | 1.03 (0.95, 1.12) | |
| **140-239:** Neoplasms | 1.06 (0.93,1.22) | 1.06 (0.93, 1.20) | |
| **240-279:** Endocrine, Nutritional and Metabolic Diseases, and Immunity Disorders | 1.10 (1.00,1.21) | 1.11 (1.01, 1.22) | |
| **280-289:** Diseases of Blood and Blood-Forming Organs | 1.15 (1.04,1.27)* | 1.14 (1.04, 1.26)* | |
| **320-389:** Diseases of the Nervous System and Sense Organs | 1.22 (1.12,1.32)*** | 1.23 (1.13, 1.33)*** | |
| **390-459:** Diseases of the Circulatory System | 1.13 (1.02,1.25)* | 1.14 (1.04, 1.26)* | |
| **460-519:** Diseases of the Respiratory System | 0.91 (0.82,1.01) | 0.94 (0.84, 1.04) | |
| **520-579:** Diseases of the Digestive System | 1.23 (1.13,1.34)*** | 1.22 (1.13, 1.33)*** | |
| **580-629:** Diseases of the Genitourinary System | 0.98 (0.89,1.07) | 0.97 (0.89, 1.05) | |
| **630-677:** Complications of Pregnancy, Childbirth, and the Puerperium | 0.92 (0.85,1.00) | 0.92 (0.85, 0.99) | |
| **680-709:** Diseases of the Skin and Subcutaneous Tissue | 1.03 (0.95,1.12) | 1.01 (0.93, 1.09) | |
| **710-739:** Diseases of the Musculoskeletal System and Connective Tissue | 1.30 (1.20,1.42)*** | 1.31 (1.21, 1.42)*** | |
| **740-759:** Congenital Anomalies | 1.14 (0.89,1.47) | 1.09 (0.85, 1.39) | |
| **760-779:** Certain Conditions Originating in the Perinatal Period | 0.98 (0.75,1.28) | 1.01 (0.78, 1.31) | |
| **780-799:** Symptoms, Signs, and Ill-Defined Conditions | 1.59 (1.42,1.78)*** | 1.59 (1.43, 1.77)*** | |
| **800-999:** Injury and Poisoning | 1.08 (0.98,1.18) | 1.08 (0.99, 1.18) | |
| **E800-E999:** Supplementary Classification of External Causes of Injury and Poisoning | 1.41 (1.14,1.73)** | 1.44 (1.18, 1.75)*** | |
| ^a^ model adjusted for SES, maternal age at delivery, total number of diagnoses during 21 months period before delivery, year of delivery, and all the somatic disorders presented in this table  *: q-value <0.05; **: q-value <0.01; ***: q-value <0.001 | | |  |

**Table S4.** Frequencies of somatic disorders by specific mental disorders

| **Somatic disorders (level 1 ICD-9 diagnostic category)** | **ICD-9:293** | | **ICD-9:300** | | **ICD-9:301** | | **ICD-9:307** | | **ICD-9:311** | |
| --- | --- | --- | --- | --- | --- | --- | --- | --- | --- | --- |
|  | **Yes** | **No** | **Yes** | **No** | **Yes** | **No** | **Yes** | **No** | **Yes** | **No** |
| **001-139:** Infectious and Parasitic Diseases | 66 | 32521 | 890 | 31697 | 43 | 32544 | 548 | 32039 | 358 | 32229 |
| **140-239:** Neoplasms | 11 | 5177 | 172 | 5016 | 10 | 5178 | 94 | 5094 | 71 | 5117 |
| **240-279:** Endocrine, Nutritional and Metabolic Diseases, and Immunity Disorders | 41 | 12041 | 447 | 11635 | 26 | 12056 | 222 | 11860 | 222 | 11860 |
| **280-289:** Diseases of Blood and Blood-Forming Organs | 37 | 10775 | 376 | 10436 | 21 | 10791 | 207 | 10605 | 162 | 10650 |
| **320-389:** Diseases of the Nervous System and Sense Organs | 60 | 26013 | 778 | 25295 | 38 | 26035 | 573 | 25500 | 304 | 25769 |
| **390-459:** Diseases of the Circulatory System | 27 | 10394 | 387 | 10034 | 19 | 10402 | 215 | 10206 | 150 | 10271 |
| **460-519:** Diseases of the Respiratory System | 93 | 50357 | 1258 | 49192 | 57 | 50393 | 769 | 49681 | 491 | 49959 |
| **520-579:** Diseases of the Digestive System | 53 | 18099 | 688 | 17464 | 29 | 18123 | 398 | 17754 | 243 | 17909 |
| **580-629:** Diseases of the Genitourinary System | 68 | 37276 | 1039 | 36305 | 57 | 37287 | 614 | 36730 | 407 | 36937 |
| **630-677:** Complications of Pregnancy, Childbirth, and the Puerperium | 60 | 30539 | 845 | 29754 | 50 | 30549 | 460 | 30139 | 349 | 30250 |
| **680-709:** Diseases of the Skin and Subcutaneous Tissue | 46 | 25860 | 737 | 25169 | 27 | 25879 | 444 | 25462 | 285 | 25621 |
| **710-739:** Diseases of the Musculoskeletal System and Connective Tissue | 64 | 22347 | 813 | 21598 | 32 | 22379 | 501 | 21910 | 275 | 22136 |
| **740-759:** Congenital Anomalies | 2 | 1124 | 36 | 1090 | 3 | 1123 | 26 | 1100 | 17 | 1109 |
| **760-779:** Certain Conditions Originating in the Perinatal Period | 1 | 1394 | 33 | 1362 | 1 | 1394 | 22 | 1373 | 12 | 1383 |
| **780-799:** Symptoms, Signs, and Ill-Defined Conditions | 103 | 44435 | 1332 | 43206 | 63 | 44475 | 798 | 43740 | 512 | 44026 |
| **800-999:** Injury and Poisoning | 26 | 12963 | 424 | 12565 | 27 | 12962 | 250 | 12739 | 146 | 12843 |
| **E800-E999:** Supplementary Classification of External Causes of Injury and Poisoning | 7 | 1432 | 57 | 1382 | 6 | 1433 | 50 | 1389 | 20 | 1419 |

Note: Cells with a frequency of less than 10 are highlighted in yellow.

**Table S5.** Associations between maternal specific mental disorders and somatic disorders

|  | **Specific mental disorders** | | | | |
| --- | --- | --- | --- | --- | --- |
|  | **Transient mental disorders due to conditions classified elsewhere  ICD-9:293** | **Anxiety, dissociative and somatoform disorders  ICD-9:300** | **Personality disorders ICD-9:301** | **Mental disorder related special symptoms or syndromes not elsewhere classified**  **ICD-9:307** | **Depressive disorder, not elsewhere classified ICD-9:311** |
| **Somatic disorders (level 1 ICD-9 diagnostic category)** | **OR (95%CI)^a^** | **OR (95%CI)^a^** | **OR (95%CI)^a^** | **OR (95%CI)^a^** | **OR (95%CI)^a^** |
| **001-139:** Infectious and Parasitic Diseases | 1.00 (0.65, 1.54) | 1.05 (0.93, 1.18) | 0.95 (0.58, 1.54) | 1.03 (0.88, 1.20) | 0.94 (0.77, 1.13) |
| **140-239:** Neoplasms | 1.35 (0.68, 2.71) | 1.13 (0.94, 1.37) | 1.39 (0.58, 3.36) | 1.03 (0.80, 1.32) | 0.95 (0.71, 1.28) |
| **240-279:** Endocrine, Nutritional and Metabolic Diseases, and Immunity Disorders | 1.33 (0.84, 2.08) | 1.09 (0.95, 1.25) | 1.43 (0.80, 2.54) | 1.05 (0.88, 1.26) | 1.32 (1.08, 1.61)** |
| **280-289:** Diseases of Blood and Blood-Forming Organs | 1.54 (0.98, 2.41) | 1.21 (1.05, 1.39)** | 1.51 (0.86, 2.66) | 1.09 (0.90, 1.30) | 1.34 (1.08, 1.66)** |
| **320-389:** Diseases of the Nervous System and Sense Organs | 1.16 (0.77, 1.75) | 1.09 (0.97, 1.23) | 1.25 (0.78, 2.02) | 1.84 (1.57, 2.15)*** | 1.07 (0.89, 1.29) |
| **390-459:** Diseases of the Circulatory System | 0.98 (0.59, 1.64) | 1.22 (1.06, 1.40)** | 1.25 (0.68, 2.31) | 1.20 (1.01, 1.44)* | 1.16 (0.93, 1.46) |
| **460-519:** Diseases of the Respiratory System | 1.02 (0.55, 1.89) | 0.95 (0.81, 1.10) | 0.52 (0.29, 0.93)* | 0.97 (0.80, 1.19) | 0.83 (0.65, 1.05) |
| **520-579:** Diseases of the Digestive System | 1.25 (0.84, 1.85) | 1.35 (1.20, 1.52)*** | 0.86 (0.51, 1.44) | 1.32 (1.13, 1.53)*** | 0.99 (0.82, 1.19) |
| **580-629:** Diseases of the Genitourinary System | 0.67 (0.44, 1.02) | 1.01 (0.89, 1.16) | 1.22 (0.66, 2.25) | 0.94 (0.80, 1.11) | 0.90 (0.73, 1.10) |
| **630-677:** Complications of Pregnancy, Childbirth, and the Puerperium | 0.78 (0.51, 1.21) | 0.97 (0.86, 1.09) | 1.23 (0.68, 2.21) | 0.88 (0.75, 1.02) | 0.90 (0.74, 1.08) |
| **680-709:** Diseases of the Skin and Subcutaneous Tissue | 0.68 (0.45, 1.03) | 1.01 (0.90, 1.14) | 0.63 (0.36, 1.10) | 1.07 (0.92, 1.25) | 0.94 (0.77, 1.14) |
| **710-739:** Diseases of the Musculoskeletal System and Connective Tissue | 1.52 (0.94, 2.46) | 1.35 (1.19, 1.52)*** | 0.80 (0.48, 1.33) | 1.43 (1.23, 1.67)*** | 0.95 (0.79, 1.15) |
| **740-759:** Congenital Anomalies | N/A | 1.01 (0.70, 1.47) | N/A | 1.04 (0.67, 1.61) | 1.34 (0.77, 2.32) |
| **760-779:** Certain Conditions Originating in the Perinatal Period | N/A | 0.88 (0.59, 1.29) | N/A | 0.91 (0.55, 1.52) | 0.78 (0.43, 1.44) |
| **780-799:** Symptoms, Signs, and Ill-Defined Conditions | 2.03 (0.97, 4.26) | 1.69 (1.42, 2.01)*** | 0.85 (0.47, 1.54) | 1.84 (1.49, 2.27)*** | 1.12 (0.88, 1.43) |
| **800-999:** Injury and Poisoning | N/A | 1.03 (0.90, 1.18) | N/A | 0.97 (0.82, 1.15) | 0.92 (0.73, 1.15) |
| **E800-E999:** Supplementary Classification of External Causes of Injury and Poisoning | 1.60 (0.72, 3.55) | 1.08 (0.80, 1.47) | 3.14 (1.23, 8.05)* | 2.22 (1.59, 3.10)*** | 0.79 (0.48, 1.28) |

^a^ Odds ratios are adjusted for SES, maternal age at delivery, total number of encounters with health services during the 21 months period before delivery, year of delivery, and all the somatic disorders presented in this table; Statistically significant positive associations are highlighted in blue and negative associations are highlighted in yellow.

*: q-value <0.05; **: q-value <0.01; ***: q-value <0.001

**Table S6.** Associations between maternal specific somatic disorders and mental disorder

| **ICD-9** | **Diagnosis / Medical code** | **OR (95%CI)^a^** | **Exposed** | |
| --- | --- | --- | --- | --- |
|  |  |  | Any mental disorder | No mental disorder |
| 8 | Intestinal infections due to other organisms | 1.39 (1.11, 1.75)* | 107 | 1136 |
| 9 | Ill-defined intestinal infections | 1.13 (0.94, 1.35) | 178 | 2466 |
| 34 | Streptococcal sore throat and scarlet fever | 0.89 (0.76, 1.03) | 262 | 4250 |
| 51 | Cowpox and paravaccinia | 2.45 (1.40, 4.31)* | 17 | 107 |
| 53 | Herpes zoster | 1.33 (0.80, 2.20) | 21 | 226 |
| 54 | Herpes simplex | 1.15 (0.95, 1.40) | 160 | 2032 |
| 57 | Other viral exanthemata | 1.09 (0.56, 2.12) | 12 | 187 |
| 70 | Viral hepatitis | 1.36 (0.78, 2.37) | 16 | 217 |
| 75 | Infectious mononucleosis | 0.99 (0.67, 1.45) | 39 | 496 |
| 77 | Other diseases of conjunctiva due to viruses and Chlamydiae | 1.00 (0.69, 1.45) | 42 | 811 |
| 78 | Other diseases due to viruses and Chlamydiae | 1.09 (0.94, 1.26) | 264 | 4237 |
| 79 | Viral and chlamydial infection in conditions classified elsewhere and of unspecified site | 0.96 (0.86, 1.06) | 621 | 9439 |
| 110 | Dermatophytosis | 1.03 (0.91, 1.17) | 413 | 6304 |
| 111 | Dermatomycosis, other and unspecified | 1.23 (0.96, 1.58) | 92 | 1211 |
| 112 | Candidiasis | 1.11 (1.00, 1.24) | 556 | 7816 |
| 127 | Other intestinal helminthiases | 1.21 (0.88, 1.65) | 57 | 656 |
| 128 | Other and unspecified helminthiases | 0.87 (0.52, 1.45) | 21 | 365 |
| 132 | Pediculosis and phthirus infestation | 0.92 (0.47, 1.79) | 12 | 147 |
| 133 | Acariasis | 0.62 (0.36, 1.09) | 16 | 285 |
| 202 | Other malignant neoplasms of lymphoid and histiocytic tissue | 2.97 (1.41, 6.25)* | 11 | 68 |
| 214 | Lipoma | 1.55 (1.01, 2.38) | 33 | 306 |
| 216 | Benign neoplasm of skin | 0.99 (0.84, 1.15) | 248 | 3674 |
| 218 | Uterine leiomyoma | 0.87 (0.52, 1.49) | 17 | 203 |
| 228 | Hemangioma and lymphangioma, any site | 1.11 (0.63, 1.94) | 18 | 193 |
| 238 | Neoplasm of uncertain behavior of other and unspecified sites and tissues | 2.39 (1.20, 4.78) | 12 | 56 |
| 240 | Simple and unspecified goiter | 1.18 (0.76, 1.83) | 30 | 314 |
| 241 | Nontoxic nodular goiter | 1.15 (0.70, 1.89) | 23 | 258 |
| 242 | Thyrotoxicosis with or without goiter | 0.91 (0.63, 1.33) | 43 | 592 |
| 244 | Acquired hypothyroidism | 0.84 (0.69, 1.02) | 158 | 2217 |
| 245 | Thyroiditis | 0.84 (0.56, 1.26) | 36 | 485 |
| 246 | Other disorders of thyroid | 1.14 (0.79, 1.64) | 42 | 460 |
| 250 | Diabetes mellitus | 1.13 (0.85, 1.50) | 68 | 734 |
| 253 | Disorders of the pituitary gland and its hypothalamic control | 1.35 (0.94, 1.95) | 40 | 426 |
| 256 | Ovarian dysfunction | 1.14 (0.83, 1.56) | 53 | 573 |
| 263 | Other and unspecified protein-calorie malnutrition | 1.32 (0.65, 2.69) | 13 | 142 |
| 266 | Deficiency of B-complex components | 1.15 (0.98, 1.35) | 244 | 2909 |
| 268 | Vitamin D deficiency | 1.31 (0.67, 2.53) | 13 | 108 |
| 269 | Other nutritional deficiencies | 1.16 (0.82, 1.65) | 48 | 561 |
| 272 | Disorders of lipoid metabolism | 1.41 (1.15, 1.72)** | 146 | 1252 |
| 275 | Disorders of mineral metabolism | 2.61 (1.60, 4.28)** | 26 | 129 |
| 276 | Disorders of fluid, electrolyte, and acid-base balance | 1.58 (1.22, 2.05)** | 87 | 746 |
| 277 | Other and unspecified disorders of metabolism | 0.96 (0.53, 1.73) | 14 | 123 |
| 278 | Overweight, obesity and other hyperalimentation | 1.24 (1.04, 1.47) | 206 | 1964 |
| 280 | Iron deficiency anemias | 1.13 (1.01, 1.27) | 493 | 6720 |
| 281 | Other deficiency anemias | 1.09 (0.80, 1.47) | 63 | 748 |
| 282 | Hereditary hemolytic anemias | 0.59 (0.33, 1.07) | 14 | 261 |
| 285 | Other and unspecified anemias | 1.41 (1.17, 1.69)** | 178 | 1910 |
| 286 | Coagulation defects | 1.10 (0.70, 1.73) | 26 | 290 |
| 287 | Purpura and other hemorrhagic conditions | 1.07 (0.72, 1.57) | 38 | 591 |
| 288 | Diseases of white blood cells | 1.27 (0.74, 2.17) | 21 | 239 |
| 289 | Other diseases of blood and blood-forming organs | 0.84 (0.60, 1.18) | 47 | 649 |
| 337 | Disorders of the autonomic nervous system | 1.49 (0.84, 2.63) | 19 | 123 |
| 345 | Epilepsy and recurrent seizures | 2.76 (1.76, 4.33)*** | 36 | 150 |
| 346 | Migraine | 2.28 (1.94, 2.68)*** | 257 | 1530 |
| 350 | Trigeminal nerve disorders | 1.89 (0.90, 3.97) | 11 | 72 |
| 351 | Facial nerve disorders | 1.07 (0.52, 2.23) | 11 | 105 |
| 353 | Nerve root and plexus disorders | 1.28 (0.70, 2.35) | 17 | 129 |
| 354 | Mononeuritis of upper limb and mononeuritis multiplex | 1.59 (1.25, 2.02)** | 106 | 813 |
| 355 | Mononeuritis of lower limb | 1.04 (0.81, 1.34) | 101 | 1568 |
| 360 | Disorders of the globe | 1.14 (0.56, 2.32) | 12 | 154 |
| 367 | Disorders of refraction and accommodation | 1.14 (0.99, 1.31) | 321 | 4477 |
| 368 | Visual disturbances | 1.67 (1.21, 2.30)* | 57 | 463 |
| 370 | Keratitis | 0.99 (0.56, 1.74) | 15 | 215 |
| 371 | Corneal opacity and other disorders of cornea | 1.02 (0.59, 1.75) | 19 | 285 |
| 372 | Disorders of conjunctiva | 0.93 (0.83, 1.05) | 464 | 7437 |
| 373 | Inflammation of eyelids | 0.79 (0.62, 0.99) | 103 | 1876 |
| 374 | Other disorders of eyelids | 0.89 (0.54, 1.47) | 22 | 363 |
| 375 | Disorders of lacrimal system | 1.51 (1.15, 1.97)* | 81 | 723 |
| 378 | Strabismus and other disorders of binocular eye movements | 1.72 (1.10, 2.67) | 30 | 289 |
| 379 | Other disorders of eye | 1.58 (1.22, 2.03)** | 91 | 918 |
| 380 | Disorders of external ear | 1.04 (0.90, 1.20) | 294 | 4572 |
| 381 | Nonsuppurative otitis media and Eustachian tube disorders | 1.19 (1.00, 1.41) | 197 | 2372 |
| 382 | Suppurative and unspecified otitis media | 1.12 (0.92, 1.36) | 146 | 1998 |
| 384 | Other disorders of tympanic membrane | 0.81 (0.45, 1.45) | 14 | 298 |
| 386 | Vertiginous syndromes and other disorders of vestibular system | 2.30 (1.73, 3.07)*** | 75 | 431 |
| 388 | Other disorders of ear | 1.26 (1.07, 1.48)* | 228 | 2597 |
| 389 | Hearing loss | 1.53 (1.18, 1.99)* | 84 | 741 |
| 401 | Essential hypertension | 1.45 (1.14, 1.86)* | 102 | 819 |
| 424 | Other diseases of endocardium | 1.95 (1.26, 3.02)* | 33 | 209 |
| 427 | Cardiac dysrhythmias | 2.80 (1.96, 4.01)*** | 48 | 238 |
| 448 | Disease of capillaries | 1.23 (0.90, 1.67) | 55 | 717 |
| 451 | Phlebitis and thrombophlebitis | 0.57 (0.35, 0.94) | 24 | 481 |
| 454 | Varicose veins of lower extremities | 0.84 (0.70, 1.00) | 201 | 3387 |
| 455 | Hemorrhoids | 1.12 (0.96, 1.31) | 243 | 3176 |
| 458 | Hypotension | 1.14 (0.84, 1.53) | 63 | 722 |
| 459 | Other disorders of circulatory system | 0.73 (0.43, 1.25) | 20 | 436 |
| 460 | Acute nasopharyngitis [common cold] | 1.16 (1.00, 1.34) | 281 | 4033 |
| 461 | Acute sinusitis | 1.06 (0.96, 1.18) | 673 | 9363 |
| 462 | Acute pharyngitis | 0.90 (0.82, 0.99) | 804 | 13260 |
| 463 | Acute tonsillitis | 0.82 (0.75, 0.91)*** | 836 | 15690 |
| 464 | Acute laryngitis and tracheitis | 1.04 (0.87, 1.25) | 192 | 2635 |
| 465 | Acute upper respiratory infections of multiple or unspecified sites | 1.01 (0.93, 1.10) | 1532 | 24504 |
| 466 | Acute bronchitis and bronchiolitis | 0.96 (0.85, 1.08) | 459 | 6875 |
| 470 | Deviated nasal septum | 1.56 (0.99, 2.45) | 26 | 250 |
| 472 | Chronic pharyngitis and nasopharyngitis | 1.13 (0.93, 1.38) | 143 | 1565 |
| 473 | Chronic sinusitis | 1.36 (0.98, 1.88) | 54 | 476 |
| 474 | Chronic disease of tonsils and adenoids | 0.88 (0.58, 1.33) | 33 | 549 |
| 476 | Chronic laryngitis and laryngotracheitis | 1.25 (0.64, 2.44) | 13 | 103 |
| 477 | Allergic rhinitis | 0.97 (0.84, 1.11) | 304 | 4045 |
| 478 | Other diseases of upper respiratory tract | 1.13 (0.98, 1.31) | 299 | 4200 |
| 483 | Pneumonia due to other specified organism | 1.19 (0.70, 2.01) | 20 | 258 |
| 485 | Bronchopneumonia, organism unspecified | 0.62 (0.32, 1.22) | 10 | 240 |
| 486 | Pneumonia, organism unspecified | 1.06 (0.78, 1.45) | 62 | 929 |
| 487 | Influenza | 0.86 (0.72, 1.04) | 168 | 2953 |
| 490 | Bronchitis, not specified as acute or chronic | 1.73 (1.15, 2.60)* | 37 | 361 |
| 493 | Asthma | 1.03 (0.85, 1.25) | 160 | 2142 |
| 521 | Diseases of hard tissues of teeth | 1.36 (1.06, 1.74) | 98 | 981 |
| 522 | Diseases of pulp and periapical tissues | 1.19 (0.98, 1.45) | 155 | 1854 |
| 523 | Gingival and periodontal diseases | 1.37 (1.04, 1.80) | 80 | 841 |
| 524 | Dentofacial anomalies, including malocclusion | 1.59 (1.22, 2.09)** | 78 | 691 |
| 525 | Other diseases and conditions of the teeth and supporting structures | 1.32 (0.92, 1.89) | 44 | 585 |
| 527 | Diseases of the salivary glands | 1.20 (0.58, 2.48) | 10 | 135 |
| 528 | Diseases of the oral soft tissues, excluding lesions specific for gingiva and tongue | 1.05 (0.88, 1.25) | 195 | 2551 |
| 529 | Diseases and other conditions of the tongue | 1.73 (1.05, 2.87) | 24 | 198 |
| 530 | Diseases of esophagus | 1.67 (1.35, 2.07)*** | 134 | 916 |
| 532 | Duodenal ulcer | 2.02 (1.30, 3.15)* | 28 | 164 |
| 533 | Peptic ulcer, site unspecified | 1.49 (0.86, 2.60) | 18 | 127 |
| 535 | Gastritis and duodenitis | 1.68 (1.42, 1.99)*** | 211 | 1639 |
| 536 | Disorders of function of stomach | 1.49 (1.14, 1.95)* | 87 | 683 |
| 540 | Acute appendicitis | 1.05 (0.61, 1.82) | 18 | 200 |
| 550 | Inguinal hernia | 1.01 (0.58, 1.74) | 17 | 269 |
| 553 | Other hernia of abdominal cavity without mention of obstruction or gangrene | 1.37 (0.97, 1.93) | 48 | 522 |
| 558 | Other and unspecified noninfectious gastroenteritis and colitis | 1.09 (0.96, 1.24) | 371 | 4477 |
| 564 | Functional digestive disorders, not elsewhere classified | 1.42 (1.21, 1.65)*** | 257 | 2347 |
| 565 | Anal fissure and fistula | 1.32 (0.98, 1.78) | 65 | 739 |
| 569 | Other disorders of intestine | 0.98 (0.70, 1.38) | 49 | 593 |
| 571 | Chronic liver disease and cirrhosis | 1.41 (0.69, 2.91) | 12 | 77 |
| 574 | Cholelithiasis | 1.02 (0.68, 1.52) | 37 | 459 |
| 575 | Other disorders of gallbladder | 1.25 (0.61, 2.56) | 10 | 131 |
| 579 | Intestinal malabsorption | 1.07 (0.54, 2.15) | 10 | 82 |
| 591 | Hydronephrosis | 1.30 (0.70, 2.41) | 15 | 157 |
| 592 | Calculus of kidney and ureter | 1.47 (0.97, 2.23) | 35 | 239 |
| 595 | Cystitis | 1.07 (0.95, 1.22) | 418 | 5788 |
| 599 | Other disorders of urethra and urinary tract | 1.07 (0.96, 1.19) | 585 | 7776 |
| 610 | Benign mammary dysplasias | 1.14 (0.90, 1.45) | 98 | 1114 |
| 611 | Other disorders of breast | 0.94 (0.80, 1.09) | 249 | 4283 |
| 614 | Inflammatory disease of ovary, fallopian tube, pelvic cellular tissue, and peritoneum | 1.51 (1.18, 1.92)** | 96 | 792 |
| 616 | Inflammatory disease of cervix, vagina, and vulva | 1.02 (0.91, 1.14) | 521 | 7411 |
| 618 | Genital prolapse | 0.98 (0.48, 2.00) | 10 | 112 |
| 620 | Noninflammatory disorders of ovary, fallopian tube, and broad ligament | 1.16 (0.89, 1.51) | 74 | 856 |
| 621 | Disorders of uterus, not elsewhere classified | 1.07 (0.55, 2.09) | 12 | 141 |
| 622 | Noninflammatory disorders of cervix | 0.95 (0.73, 1.22) | 81 | 1084 |
| 623 | Noninflammatory disorders of vagina | 0.94 (0.82, 1.07) | 336 | 4634 |
| 625 | Pain and other symptoms associated with female genital organs | 0.96 (0.78, 1.18) | 134 | 1665 |
| 626 | Disorders of menstruation and other abnormal bleeding from female genital tract | 1.04 (0.95, 1.14) | 942 | 13874 |
| 627 | Menopausal and postmenopausal disorders | 1.26 (0.71, 2.21) | 18 | 192 |
| 628 | Infertility, female | 0.80 (0.70, 0.90)** | 374 | 6058 |
| 629 | Other disorders of female genital organs | 0.84 (0.53, 1.33) | 26 | 294 |
| 632 | Missed abortion | 0.75 (0.63, 0.90)* | 179 | 3081 |
| 633 | Ectopic pregnancy | 0.91 (0.65, 1.28) | 52 | 718 |
| 634 | Spontaneous abortion | 0.85 (0.69, 1.03) | 138 | 2045 |
| 635 | Legally induced abortion | 0.63 (0.32, 1.25) | 11 | 164 |
| 640 | Hemorrhage in early pregnancy | 1.01 (0.87, 1.17) | 270 | 3872 |
| 641 | Antepartum hemorrhage, abruptio placentae, and placenta previa | 0.36 (0.17, 0.78)* | 10 | 332 |
| 642 | Hypertension complicating pregnancy, childbirth, and the puerperium | 1.16 (0.93, 1.45) | 116 | 1372 |
| 643 | Excessive vomiting in pregnancy | 1.20 (1.00, 1.45) | 167 | 1848 |
| 644 | Early or threatened labor | 1.00 (0.84, 1.20) | 182 | 2290 |
| 645 | Late pregnancy | 0.85 (0.58, 1.24) | 37 | 801 |
| 646 | Other complications of pregnancy, not elsewhere classified | 0.99 (0.85, 1.16) | 237 | 3474 |
| 648 | Other current conditions in the mother classifiable elsewhere, but complicating pregnancy, childbirth, or the puerperium | 1.02 (0.92, 1.13) | 620 | 9097 |
| 650 | Normal delivery | 0.52 (0.29, 0.92) | 16 | 528 |
| 651 | Multiple gestation | 0.99 (0.77, 1.28) | 84 | 1152 |
| 652 | Malposition and malpresentation of fetus | 0.80 (0.49, 1.30) | 21 | 365 |
| 654 | Abnormality of organs and soft tissues of pelvis | 0.98 (0.74, 1.29) | 81 | 1624 |
| 655 | Known or suspected fetal abnormality affecting management of mother | 1.28 (0.70, 2.33) | 15 | 132 |
| 656 | Other known or suspected fetal and placental problems affecting management of mother | 1.28 (0.89, 1.85) | 39 | 514 |
| 657 | Polyhydramnios | 0.96 (0.63, 1.46) | 32 | 512 |
| 658 | Other problems associated with amniotic cavity and membranes | 0.96 (0.65, 1.41) | 34 | 618 |
| 659 | Other indications for care or intervention related to labor and delivery, not elsewhere classified | 0.84 (0.56, 1.26) | 37 | 636 |
| 663 | Umbilical cord complications | 0.69 (0.46, 1.04) | 34 | 771 |
| 669 | Other complications of labor and delivery, not elsewhere classified | 0.87 (0.58, 1.29) | 34 | 576 |
| 671 | Venous complications in pregnancy and the puerperium | 0.73 (0.61, 0.87)** | 192 | 3861 |
| 675 | Infections of the breast and nipple associated with childbirth | 0.48 (0.27, 0.85) | 14 | 425 |
| 676 | Other disorders of the breast associated with childbirth and disorders of lactation | 1.26 (0.87, 1.81) | 43 | 568 |
| 680 | Carbuncle and furuncle | 1.07 (0.77, 1.47) | 55 | 704 |
| 681 | Cellulitis and abscess of finger and toe | 1.12 (0.87, 1.45) | 90 | 1317 |
| 682 | Other cellulitis and abscess | 1.07 (0.88, 1.31) | 153 | 2111 |
| 683 | Acute lymphadenitis | 0.97 (0.61, 1.54) | 24 | 396 |
| 684 | Impetigo | 1.09 (0.71, 1.66) | 32 | 468 |
| 686 | Other local infections of skin and subcutaneous tissue | 1.03 (0.78, 1.36) | 76 | 1043 |
| 690 | Erythematosquamous dermatosis | 1.25 (0.94, 1.66) | 70 | 881 |
| 691 | Atopic dermatitis and related conditions | 0.83 (0.65, 1.06) | 93 | 1669 |
| 692 | Contact dermatitis and other eczema | 0.92 (0.82, 1.04) | 449 | 7452 |
| 695 | Erythematous conditions | 1.13 (0.82, 1.56) | 53 | 738 |
| 696 | Psoriasis and similar disorders | 0.80 (0.59, 1.09) | 60 | 1118 |
| 698 | Pruritus and related conditions | 1.09 (0.92, 1.29) | 192 | 2390 |
| 700 | Corns and callosities | 0.90 (0.64, 1.25) | 57 | 973 |
| 701 | Other hypertrophic and atrophic conditions of skin | 1.31 (1.04, 1.65) | 109 | 1279 |
| 702 | Other dermatoses | 0.70 (0.43, 1.15) | 20 | 427 |
| 703 | Diseases of nail | 1.07 (0.84, 1.37) | 99 | 1604 |
| 704 | Diseases of hair and hair follicles | 1.26 (1.05, 1.51) | 180 | 1965 |
| 705 | Disorders of sweat glands | 1.02 (0.70, 1.49) | 37 | 579 |
| 706 | Diseases of sebaceous glands | 1.22 (1.07, 1.39)* | 368 | 4965 |
| 708 | Urticaria | 0.95 (0.76, 1.17) | 118 | 1697 |
| 709 | Other disorders of skin and subcutaneous tissue | 0.96 (0.77, 1.19) | 119 | 1670 |
| 710 | Diffuse diseases of connective tissue | 1.06 (0.54, 2.08) | 11 | 151 |
| 713 | Arthropathy associated with other disorders classified elsewhere | 1.97 (1.14, 3.43) | 20 | 144 |
| 714 | Rheumatoid arthritis and other inflammatory polyarthropathies | 1.44 (0.80, 2.60) | 18 | 138 |
| 715 | Osteoarthrosis and allied disorders | 1.15 (0.61, 2.15) | 15 | 141 |
| 716 | Other and unspecified arthropathies | 1.04 (0.55, 1.98) | 14 | 167 |
| 717 | Internal derangement of knee | 1.31 (0.92, 1.88) | 43 | 498 |
| 719 | Other and unspecified disorders of joint | 1.30 (1.14, 1.49)** | 355 | 3625 |
| 720 | Ankylosing spondylitis and other inflammatory spondylopathies | 2.08 (1.03, 4.22) | 11 | 77 |
| 721 | Spondylosis and allied disorders | 1.47 (0.76, 2.86) | 13 | 98 |
| 722 | Intervertebral disc disorders | 1.51 (1.05, 2.17) | 42 | 304 |
| 723 | Other disorders of cervical region | 1.73 (1.51, 1.98)*** | 345 | 2636 |
| 724 | Other and unspecified disorders of back | 1.38 (1.25, 1.51)*** | 936 | 10254 |
| 726 | Peripheral enthesopathies and allied syndromes | 1.03 (0.86, 1.23) | 190 | 2433 |
| 727 | Other disorders of synovium, tendon, and bursa | 1.06 (0.82, 1.36) | 94 | 1190 |
| 728 | Disorders of muscle, ligament, and fascia | 1.34 (1.02, 1.77) | 79 | 700 |
| 729 | Other disorders of soft tissues | 1.30 (1.16, 1.46)*** | 522 | 5510 |
| 733 | Other disorders of bone and cartilage | 1.94 (1.34, 2.82)** | 48 | 320 |
| 734 | Flat foot | 0.91 (0.63, 1.31) | 46 | 643 |
| 736 | Other acquired deformities of limbs | 2.06 (1.07, 3.94) | 13 | 100 |
| 737 | Curvature of spine | 1.56 (0.96, 2.54) | 23 | 254 |
| 754 | Certain congenital musculoskeletal deformities | 1.38 (0.76, 2.51) | 15 | 184 |
| 757 | Congenital anomalies of the integument | 1.37 (0.89, 2.11) | 27 | 309 |
| 764 | Slow fetal growth and fetal malnutrition | 0.99 (0.70, 1.41) | 43 | 792 |
| 766 | Disorders relating to long gestation and high birthweight | 0.74 (0.38, 1.46) | 11 | 212 |
| 780 | General symptoms | 1.60 (1.46, 1.74)*** | 1284 | 14036 |
| 781 | Symptoms involving nervous and musculoskeletal systems | 3.11 (1.92, 5.05)*** | 25 | 127 |
| 782 | Symptoms involving skin and other integumentary tissue | 1.17 (1.00, 1.36) | 280 | 3670 |
| 783 | Symptoms concerning nutrition, metabolism, and development | 1.97 (1.57, 2.46)*** | 124 | 854 |
| 784 | Symptoms involving head and neck | 1.61 (1.47, 1.76)*** | 1118 | 11152 |
| 785 | Symptoms involving cardiovascular system | 2.10 (1.83, 2.40)*** | 373 | 2365 |
| 786 | Symptoms involving respiratory system and other chest symptoms | 1.27 (1.16, 1.40)*** | 848 | 9644 |
| 787 | Symptoms involving digestive system | 1.49 (1.32, 1.67)*** | 491 | 4512 |
| 788 | Symptoms involving urinary system | 1.14 (0.99, 1.32) | 283 | 3106 |
| 789 | Other symptoms involving abdomen and pelvis | 1.23 (1.12, 1.34)*** | 1279 | 16341 |
| 790 | Nonspecific findings on examination of blood | 1.09 (0.87, 1.36) | 125 | 1477 |
| 791 | Nonspecific findings on examination of urine | 0.72 (0.42, 1.22) | 19 | 310 |
| 794 | Nonspecific abnormal results of function studies | 0.65 (0.35, 1.20) | 14 | 235 |
| 795 | Other and nonspecific abnormal cytological, histological, immunological and DNA test findings | 0.61 (0.30, 1.25) | 11 | 202 |
| 799 | Other ill-defined and unknown causes of morbidity and mortality | 1.26 (1.14, 1.40)*** | 703 | 8649 |
| 813 | Fracture of radius and ulna | 0.94 (0.48, 1.85) | 12 | 141 |
| 816 | Fracture of one or more phalanges of hand | 1.05 (0.55, 2.02) | 11 | 127 |
| 824 | Fracture of ankle | 1.69 (0.85, 3.38) | 12 | 129 |
| 825 | Fracture of one or more tarsal and metatarsal bones | 1.23 (0.67, 2.28) | 13 | 176 |
| 845 | Sprains and strains of ankle and foot | 1.02 (0.79, 1.30) | 94 | 1428 |
| 847 | Sprains and strains of other and unspecified parts of back | 1.54 (1.22, 1.95)** | 101 | 765 |
| 854 | Intracranial injury of other and unspecified nature | 3.39 (1.97, 5.82)*** | 20 | 83 |
| 873 | Other open wound of head | 0.76 (0.39, 1.50) | 10 | 153 |
| 879 | Open wound of other and unspecified sites, except limbs | 0.96 (0.46, 2.00) | 11 | 173 |
| 883 | Open wound of finger(s) | 0.88 (0.59, 1.33) | 31 | 493 |
| 910 | Superficial injury of face, neck, and scalp except eye | 1.22 (0.89, 1.68) | 59 | 741 |
| 918 | Superficial injury of eye and adnexa | 1.15 (0.64, 2.07) | 14 | 239 |
| 919 | Superficial injury of other, multiple, and unspecified sites | 1.13 (0.78, 1.65) | 38 | 514 |
| 920 | Contusion of face, scalp, and neck except eye(s) | 1.56 (0.99, 2.47) | 26 | 229 |
| 922 | Contusion of trunk | 1.45 (1.14, 1.84)* | 99 | 824 |
| 923 | Contusion of upper limb | 1.19 (0.92, 1.54) | 88 | 973 |
| 924 | Contusion of lower limb and of other and unspecified sites | 1.26 (1.03, 1.53) | 145 | 1614 |
| 945 | Burn of lower limb(s) | 2.05 (1.05, 4.00) | 14 | 109 |
| 949 | Burn, unspecified | 1.12 (0.78, 1.60) | 43 | 538 |
| 958 | Certain early complications of trauma | 0.95 (0.61, 1.47) | 32 | 518 |
| 959 | Injury, other and unspecified | 1.06 (0.68, 1.67) | 27 | 371 |
| 989 | Toxic effect of other substances, chiefly nonmedicinal as to source | 1.14 (0.56, 2.32) | 10 | 149 |
| 994 | Effects of other external causes | 4.11 (1.96, 8.63)** | 13 | 48 |
| 995 | Certain adverse effects not elsewhere classified | 0.90 (0.75, 1.08) | 163 | 2219 |
| E81 | Motor vehicle traffic accidents | 1.64 (1.23, 2.18)** | 70 | 516 |
| E92 | Late effects of accidental injury | 1.42 (0.98, 2.07) | 42 | 398 |
| E96 | Homicide and injury purposely inflicted by other persons | 1.16 (0.79, 1.72) | 34 | 411 |

^a^ Odds ratios are adjusted for SES, maternal age at delivery, total number of encounters with health services during 21 months period before delivery, and year of delivery

*: Q-value <0.05; **: Q-value <0.01; ***: Q-value <0.001

**Table S7.** Associations between maternal mental disorder and specific somatic disorders

| **ICD-9 level 1** | **ICD-9 level 2** | **ICD-9 level 3** | **Univariate adjusted model^a^** | **Multivariable model^b^** | **Exposed** | |
| --- | --- | --- | --- | --- | --- | --- |
|  |  |  | **OR (95%CI)** | **OR (95%CI)** | Any mental disorder | No mental disorder |
| **Infectious and Parasitic Diseases** | Intestinal Infectious Diseases | 8 | 1.39 (1.11, 1.75)* | 1.28 (1.01, 1.62)* | 107 | 1136 |
|  | Viral Diseases Generally Accompanied by Exanthem | 51 | 2.45 (1.40, 4.31)* | 2.61 (1.48, 4.60)*** | 17 | 107 |
| **Neoplasms** | Neoplasms of Uncertain Behavior | 202 | 2.97 (1.41, 6.25)* | 3.30 (1.53, 7.11)** | 11 | 68 |
| **Endocrine, Nutritional and Metabolic Diseases, and Immunity Disorders** | Other Metabolic and Immunity Disorders | 272 | 1.41 (1.15, 1.72)** | 1.26 (1.02, 1.55)* | 146 | 1252 |
|  |  | 275 | 2.61 (1.60, 4.28)** | 2.44 (1.49, 3.98)*** | 26 | 129 |
|  |  | 276 | 1.58 (1.22, 2.05)** | 1.22 (0.93, 1.61) | 87 | 746 |
| **Diseases of the Blood and Blood-Forming Organs** | Diseases of the Blood and Blood-Forming Organs | 285 | 1.41 (1.17, 1.69)** | 1.29 (1.07, 1.56)** | 178 | 1910 |
| **Diseases of the Nervous System and Sense Organs** | Other Disorders of the Central Nervous System | 345 | 2.76 (1.76, 4.33)*** | 3.06 (1.93, 4.86)*** | 36 | 150 |
|  |  | 346 | 2.28 (1.94, 2.68)*** | 1.91 (1.61, 2.27)*** | 257 | 1530 |
|  | Disorders of the Peripheral Nervous System | 354 | 1.59 (1.25, 2.02)** | 1.35 (1.05, 1.73)* | 106 | 813 |
|  | Disorders of the Eye and Adnexa | 368 | 1.67 (1.21, 2.30)* | 1.28 (0.92, 1.78) | 57 | 463 |
|  |  | 375 | 1.51 (1.15, 1.97)* | 1.39 (1.06, 1.83)* | 81 | 723 |
|  |  | 379 | 1.58 (1.22, 2.03)** | 1.37 (1.05, 1.79)* | 91 | 918 |
|  | Diseases of the Ear and Mastoid Process | 386 | 2.30 (1.73, 3.07)*** | 1.71 (1.25, 2.33)*** | 75 | 431 |
|  |  | 388 | 1.26 (1.07, 1.48)* | 1.06 (0.89, 1.25) | 228 | 2597 |
|  |  | 389 | 1.53 (1.18, 1.99)* | 1.34 (1.03, 1.74)* | 84 | 741 |
| **Diseases of the Circulatory System** | Hypertensive Disease | 401 | 1.45 (1.14, 1.86)* | 1.37 (1.07, 1.76)* | 102 | 819 |
|  | Other Forms of Heart Disease | 424 | 1.95 (1.26, 3.02)* | 1.26 (0.79, 2.00) | 33 | 209 |
|  | Other Forms of Heart Disease | 427 | 2.80 (1.96, 4.01)*** | 2.03 (1.39, 2.98)*** | 48 | 238 |
| **Diseases of the Respiratory System** | Acute Respiratory Infections | 463 | 0.82 (0.75, 0.91)*** | 0.78 (0.71, 0.86)*** | 836 | 15690 |
|  | Chronic Obstructive Pulmonary Disease and Allied Conditions | 490 | 1.73 (1.15, 2.60)* | 1.62 (1.06, 2.47)* | 37 | 361 |
| **Diseases of the Digestive System** | Diseases of Oral Cavity, Salivary Glands, and Jaws | 524 | 1.59 (1.22, 2.09)** | 1.32 (0.99, 1.76) | 78 | 691 |
|  | Diseases of Esophagus, Stomach, and Duodenum | 530 | 1.67 (1.35, 2.07)*** | 1.23 (0.97, 1.57) | 134 | 916 |
|  |  | 532 | 2.02 (1.30, 3.15)* | 1.56 (0.93, 2.60) | 28 | 164 |
|  |  | 535 | 1.68 (1.42, 1.99)*** | 1.29 (1.07, 1.56)** | 211 | 1639 |
|  |  | 536 | 1.49 (1.14, 1.95)* | 1.09 (0.82, 1.44) | 87 | 683 |
|  | Other Diseases of Intestines and Peritoneum | 564 | 1.42 (1.21, 1.65)*** | 1.23 (1.05, 1.45)* | 257 | 2347 |
| **Diseases of the Genitourinary System** | Inflammatory Disease of Female Pelvic Organs | 614 | 1.51 (1.18, 1.92)** | 1.38 (1.06, 1.79)* | 96 | 792 |
|  | Other Disorders of Female Genital Tract | 628 | 0.80 (0.70, 0.90)** | 0.86 (0.76, 0.98)* | 374 | 6058 |
| **Complications of Pregnancy, Childbirth, and the Puerperium** | Ectopic And Molar Pregnancy and Other Pregnancy with Abortive Outcome | 632 | 0.75 (0.63, 0.90)* | 0.83 (0.69, 1.00)* | 179 | 3081 |
|  | Complications Mainly Related to Pregnancy | 641 | 0.36 (0.17, 0.78)* | 0.37 (0.17, 0.80)* | 10 | 332 |
|  | Complications of the Puerperium | 671 | 0.73 (0.61, 0.87)** | 0.76 (0.64, 0.92)** | 192 | 3861 |
| **Diseases Of the Skin and Subcutaneous Tissue** | Other Diseases of Skin and Subcutaneous Tissue | 706 | 1.22 (1.07, 1.39)* | 1.18 (1.03, 1.34)* | 368 | 4965 |
| **Diseases of the Musculoskeletal System and Connective Tissue** | Arthropathies and Related Disorders | 719 | 1.30 (1.14, 1.49)** | 1.10 (0.95, 1.26) | 355 | 3625 |
|  | Dorsopathies | 723 | 1.73 (1.51, 1.98)*** | 1.32 (1.14, 1.53)*** | 345 | 2636 |
|  |  | 724 | 1.38 (1.25, 1.51)*** | 1.20 (1.09, 1.32)*** | 936 | 10254 |
|  | Rheumatism, Excluding the Back | 729 | 1.30 (1.16, 1.46)*** | 1.06 (0.94, 1.20) | 522 | 5510 |
|  | Osteopathies, Chondropathies, and Acquired Musculoskeletal Deformities | 733 | 1.94 (1.34, 2.82)** | 1.56 (1.06, 2.30)* | 48 | 320 |
| **Symptoms, Signs, and Ill-Defined Conditions** | Symptoms | 780 | 1.60 (1.46, 1.74)*** | 1.38 (1.26, 1.51)*** | 1284 | 14036 |
|  |  | 781 | 3.11 (1.92, 5.05)*** | 2.50 (1.46, 4.27)*** | 25 | 127 |
|  |  | 783 | 1.97 (1.57, 2.46)*** | 1.67 (1.32, 2.11)*** | 124 | 854 |
|  |  | 784 | 1.61 (1.47, 1.76)*** | 1.36 (1.23, 1.49)*** | 1118 | 11152 |
|  |  | 785 | 2.10 (1.83, 2.40)*** | 1.71 (1.47, 1.97)*** | 373 | 2365 |
|  |  | 786 | 1.27 (1.16, 1.40)*** | 1.09 (0.99, 1.21) | 848 | 9644 |
|  |  | 787 | 1.49 (1.32, 1.67)*** | 1.18 (1.03, 1.34)* | 491 | 4512 |
|  |  | 789 | 1.23 (1.12, 1.34)*** | 1.03 (0.94, 1.13) | 1279 | 16341 |
|  | Ill-Defined and Unknown Causes of Morbidity and Mortality | 799 | 1.26 (1.14, 1.40)*** | 1.13 (1.02, 1.26)* | 703 | 8649 |
| **Injury and Poisoning** | Sprains and Strains of Joints and Adjacent Muscles | 847 | 1.54 (1.22, 1.95)** | 1.03 (0.76, 1.41) | 101 | 765 |
|  | Intracranial Injury, Excluding Those with Skull Fracture | 854 | 3.39 (1.97, 5.82)*** | 1.90 (1.04, 3.46)* | 20 | 83 |
|  | Contusion with Intact Skin Surface | 922 | 1.45 (1.14, 1.84)* | 1.11 (0.84, 1.46) | 99 | 824 |
|  | Other and Unspecified Effects of External Causes | 994 | 4.11 (1.96, 8.63)** | 3.82 (1.73, 8.42)*** | 13 | 48 |
| **Supplementary Classification of External Causes of Injury and Poisoning** | Motor Vehicle Traffic Accidents | E81 | 1.64 (1.23, 2.18)** | 1.27 (0.86, 1.88) | 70 | 516 |

Note: Statistically significant positive associations from the multivariable model are highlighted in blue and negative associations are highlighted in yellow.
^a^ models adjusted for SES, maternal age at delivery, total number of encounters with health services during the 21 months period before delivery, and year of delivery ^b^ models adjusted for SES, maternal age at delivery, total number of encounters with health services during the 21 months period before delivery, year of delivery, and all the specific somatic disorders presented in this table
*: q-value <0.05; **: q-value <0.01; ***: q-value <0.001
